# Sleep, physical activity, sedentary behavior, and risk of cataract: a cross-sectional and prospective study from UK Biobank

**DOI:** 10.1186/s12916-025-04312-7

**Published:** 2025-08-08

**Authors:** Yu Peng, Yuzhou Zhang, Ka Wai Kam, Mary Ho, Sunny Au, Xiujuan Zhang, Mandy PH Ng, Patrick Ip, Alvin Young, Chi Pui Pang, Clement C. Tham, Li Jia Chen, Jason C. Yam

**Affiliations:** 1 Department of Ophthalmology and Visual Sciences, The Chinese University of Hong Kong, Hong Kong SAR, China; 2 Department of Ophthalmology and Visual Sciences, Prince of Wales Hospital, Hong Kong SAR, China; 3 Department of Ophthalmology, Tung Wah Eastern Hospital, Causeway bay, Hong Kong SAR, China; 4 Department of Ophthalmology, The University of Hong Kong, Hong Kong SAR, China; 5 Department of Paediatrics and Adolescent Medicine, Li Ka Shing Faculty of Medicine, The University of Hong Kong, Hong Kong SAR, China; 6 Hong Kong Hub of Paediatric Excellence, The Chinese University of Hong Kong, Hong Kong SAR, China; 7 Joint Shantou International Eye Center of Shantou University and The Chinese University of Hong Kong, Shantou, China; 8 Hong Kong Eye Hospital, Hong Kong SAR, China; 9 Department of Ophthalmology, Hong Kong Children’s Hospital, Hong Kong SAR, China

**Keywords:** **S**leep, Physical activity, Sedentary behavior, Cataract, UK Biobank

## Abstract

**Background:**

Cataract is a leading cause of visual impairment globally. Although daily behaviors such as sleep, physical activity (PA), and sedentary behavior (SB) have been associated with cataract risk, the evidence remains controversial and uncertain. This study aimed to examine the independent and combined effects of sleep, PA, and SB on cataract risk using data from the UK Biobank.

**Methods:**

The cross-sectional analyses included 440,645 participants from the UK Biobank with complete data on sleep, PA, and SB. For the longitudinal analyses, a subset of 426,540 participants without cataract at baseline was included. Cataract cases were identified through hospital inpatient records and self-reported data. Baseline data on sleep, PA, and SB were collected via touchscreen questionnaires. Logistic regression and Cox proportional hazards models were used to examine the independent and synergistic associations between sleep, PA, SB, and cataract.

**Results:**

In the cross-sectional analyses, 440,645 participants were evaluated (54.0% female; mean [SD] age, 56.5 [8.1] years), of whom 14,105 (3.2%) had cataract. Significant associations were found between poor sleep (OR, 1.35; 95% CI, 1.23–1.48), low PA (OR, 1.06; 95% CI, 1.01–1.11), and cataract. Longitudinal analyses included 426,540 participants (53.9% female; mean [SD] age, 56.3 [8.1] years). During a mean follow-up period of 10.8 years, 55,658 incident cataract cases were recorded. Poor sleep (HR, 1.14; 95% CI, 1.08–1.19), low PA (HR, 1.05; 95% CI, 1.02–1.07), and high SB (HR, 1.08; 95% CI, 1.06–1.11) were correlated with increased cataract risk. The combination of poor sleep, low PA, and high SB further elevated the risk, with the highest odds in cross-sectional (OR, 1.73; 95% CI, 1.37–2.15) and prospective (HR, 1.37; 95% CI, 1.21–1.55) analyses. Replacing 1 h/day of SB time with an equal time spent in total PA and sleep was associated with a 1.7% and 2.7% decreased risk of cataract, respectively.

**Conclusions:**

Poor sleep, low PA, and high SB are independently and jointly associated with an increased risk of cataract. Modifying these behaviors, either individually or in combination, can effectively mitigate the risk of cataract.

**Supplementary Information:**

The online version contains supplementary material available at 10.1186/s12916-025-04312-7.

## Background

It is estimated that approximately 94 million people worldwide suffer from blindness or visual impairment, with cataract remaining the predominant cause of visual loss [1–3]. The impact of cataract on visual impairment can significantly affect an individual’s overall quality of life, particularly among the elderly population [4–6]. The development of cataract is influenced by various factors that contribute to the aging of the lens. Although advancing age stands as the primary and unchangeable factor in cataract development, multiple modifiable risk factors have been recognized, such as prolonged exposure to ultraviolet light, smoking, alcohol consumption, and diabetes [7–14].

Lifestyle-based approaches are gaining increasing attention in cataract management. Sleep, physical activity (PA), and sedentary behavior (SB) are three modifiable daily lifestyle factors that have been independently associated with cataract risk in previous studies [15–20]. However, existing findings were frequently based on cohorts with limited sample sizes and showed inconsistencies. For instance, while sleep apnea has been reported to elevate cataract risk [15], Chen et al. found no significant association between cataract incidence and sleep quality [16]. Similarly, some studies suggested that low PA and high SB were correlated with increased cataract risk [18, 21], whereas the Beijing Eye Study reported no significant relationship between cataract occurrence and either PA levels or sedentary lifestyles [19]. Given the growing emphasis on movement behaviors in ocular health, further epidemiological investigations with larger cohorts are warranted to clarify the relationships among sleep, PA, SB, and cataract.

Additionally, these behaviors are closely interrelated in daily life. Prolonged sedentary behavior may limit opportunities for PA engagement, while poor sleep quality can reduce energy reserves necessary for physical exertion, creating a cyclical relationship that potentiates adverse health outcomes. Despite evidence has indicated that multi-behavioral interventions can yield greater health benefits than single-factor approaches [22], no study to date has investigated the synergistic effects of sleep, PA, and SB on cataract development. Gaining insight into these interactions could aid in creating lifestyle interventions for the prevention and management of cataract.

Therefore, the objective of this study is to investigate the independent and joint effects of sleep, PA and SB on the risk of cataract using cross-sectional and longitudinal data from the UK Biobank.

## Methods

### Study population

Information on the design and methodology of the UK Biobank study has been previously documented [23]. Briefly, the UK Biobank is a large prospective cohort study involving more than 500,000 individuals aged 37 to 73 years from 22 assessment sites across the UK. At recruitment, data on socio-demographic factors, lifestyles, and medical histories were collected through touchscreen questionnaires. Additionally, physical measurements were conducted during the initial assessment stage. Participants were followed up through regular online questionnaire assessments and linkage to electronic health records, including primary care data, hospital admissions, and national cancer and mortality registries. Ethical approval of UK biobank study was obtained from the North West Multicenter Research Ethics Committee and every participant involved provided written informed consent. This study has been conducted using the UK Biobank Resource under Application Number 91320.

In this study, individuals with missing data on sleep, PA, and SB were initially excluded (n = 55,072). Furthermore, participants with erroneous records were subsequently excluded (n = 6,684). A total of 440,645 individuals were included for cross-sectional analyses. For longitudinal analyses, participants with existing cataract at baseline were also excluded (n = 14,105), resulting in a final sample size of 426,540 individuals. The flowchart of study participants is presented in Fig. S1 (Additional file 1).

### Outcome ascertainment

Cataract events were identified using International Classification of Diseases (ICD) codes, Classification of Surgical Operations and Procedures (OPCS-4), and self-reported information. The corresponding codes and self-reported field codes are listed in Table S1 (Additional file 2).

### Sleep, physical activity, and sedentary behavior measurements

The sleep score was calculated based on five sleep characteristics: chronotype, sleep duration, insomnia, snoring, and daytime sleepiness [24–28]. Details on these characteristics are listed in Table S2 (Additional file 2). Participants were assigned a score ranging from 0 to 5, based on the presence of healthy sleep characteristics. Subsequently, participants were categorized into three groups: healthy sleep (sleep score of 4–5), intermediate sleep (sleep score of 2–3), and poor sleep (sleep score of 0–1) [24, 26].

PA was evaluated through the frequency and duration of walking (Field 864 and 874), moderate activity (Field 884 and 894), and vigorous activity (Field 904 and 914), utilizing a modified version of the International Physical Activity Questionnaire (IPAQ) [29]. The combination of these measures involved multiplying the metabolic equivalents (METs) assigned to each type of PA by the total minutes engaged in each activity per week. The calculation of a continuous PA score in MET-minutes per week was done by assigning 3.3 METs to walking, 4 METs to moderate activity, and 8 METs to vigorous activity [30]. In accordance with the IPAQ [29], any PA duration less than 10 min per day for any category was recoded as 0. Furthermore, any time variables exceeding four hours for walking, moderate activity, and vigorous activity were truncated and set equal to 240 min. Participants exceeding 16 h per day in any type of PA duration were excluded from the analysis due to the data being deemed unreasonably high [30]. Based on the World Health Organization (WHO) PA guideline [29], PA was categorized as low (0 to < 600 MET-mins/week), moderate (600 to < 1200 MET-mins/week) and high (≥ 1200 MET-mins/week).

During their initial visit to the assessment center, participants reported the number of hours spent daily watching television, using computers (excluding work-related use), and driving (Fields 1070, 1080, and 1090). The cumulative sum of these hours indicated the total SB time for each individual [30–33]. Subsequently, participants were classified into three groups according to their SB tertiles: low (0–4 h/day), moderate (4–5 h/day), and high (≥ 6 h/day). Participants whose total sum of sedentary time, sleep duration, and PA exceeded 24 h were excluded from the analyses.

To address the concern about the small sample sizes within specific subgroups while investigating the combined effects of three exposures and cataract, and focusing on high-risk behavioral clusters, we followed Huang et al.'s approach [30], and reclassified sleep, PA, and SB into binary categories as needed. Sleep was categorized as good sleep (sleep score of ≥ 2) or poor sleep (sleep score of 0–1). PA was divided into two groups: non-ideal PA (< 600 MET-mins/week) and ideal PA (≥ 600 MET-mins/week). SB was reclassified as low to moderate SB (< 6 h/day) and high SB (≥ 6 h/day).

### Covariates

The confounding variables were chosen based on published research evidence from existing literature [34–36]. The confounding variables included age, sex, ethnicity, socioeconomic status, body mass index (BMI), education, smoking and drinking status, sun exposure, diabetes and hypertension history. Detailed definitions of these variables were described in Table S3 (Additional file 2).

## Statistical analyses

The baseline characteristics of UK Biobank participants were presented, stratified based on their cataract status. Multivariable logistic regression analyses were performed to evaluate the cross-sectional relationship between sleep, PA, SB, and cataract at baseline. Cox proportional hazards regression models were conducted to examine the longitudinal associations between sleep, PA, SB, and the occurrence of cataract. The onset date of cataract was determined as the earliest occurrence date. The time period was defined as the interval between the initial enrollment date to the occurrence of cataract, loss to follow-up, mortality, or the censoring date (December 31, 2020, for England and Wales, and January 18, 2021, for Scotland), whichever came first. Initially, each analysis commenced with a foundational model incorporating age and sex as covariates (Model 1). Subsequently, a secondary model (Model 2) was used by supplementing the initial variables with socioeconomic status, ethnicity, educational levels, BMI, drinking and smoking status, sun exposure, diabetes, and hypertension. We performed restricted cubic spline (RCS) analyses with three knots to assess the dose–response relationship between sleep, PA, sedentary time and cataract risk. To delve deeper into the potential combined impacts of the three exposures, supplementary analyses were conducted to examine additive interactions among these variables.

We further adopted an isotemporal substitution model to evaluate the effect of replacing SB with equal time of PA or sleep on cataract risk. This approach is based on the realistic assumption that an increase in one behavior will be accompanied by a decrease of equivalent duration (isotemporal) in another behavior, thereby maintaining a constant total time across all behaviors [37]. This model could be expressed as:

$$h\;(t)\;=\;h_0\;(t)\;\exp\;(\beta_1\;\times\;\mathrm{PA}\;+\;\beta_2\;\times\;\mathrm{sleep}\;+\;\beta_3\mathit\;\times\mathit\;\mathrm{total}\;\mathrm{time}\;+\;\beta_4\mathit\;\times\mathit\;\mathrm{covariates}).$$

The total time = SB time + total PA time + sleep duration. The β_1_–β_2_ represents the effects of substituting 1 h/day SB with 1 h/day PA, and sleep, respectively.

Additionally, subgroup analyses were performed, stratified by age (< 60 and ≥ 60 years), sex (male and female), and diabetes status (yes and no), aiming to investigate potential variations within these subgroups. A likelihood ratio test was conducted to evaluate the potential interactions among the variables. Furthermore, a series of sensitivity analyses were performed to ensure the robustness of the findings. First, to mitigate concerns related to reverse causation, the analyses were repeated with the exclusion of the first two years of follow-up duration. Second, mutual adjustments for sleep, PA, and SB were applied according to Model 2. Third, additional adjustments were made for eye trauma, myopia, and corticosteroid use based on Model 2. Fourth, to minimize potential recall bias and measurement errors associated with questionnaire-derived data, the primary analysis was repeated within a subsample using data obtained from Axivity AX3 wrist-worn triaxial accelerometers. Additional information on accelerometer-derived data collection and processing was reported in the Supplementary Methods (Additional file 2) and Fig. S2 (Additional file 1) [38].

All analyses were carried out using R software version 4.3.1. A 2-sided *P*-value of less than 0.05 was considered statistically significant.

## Results

### Baseline characteristics of the participates

Table 1 presents the baseline characteristics of the participants, categorized based on their cataract status in both the cross-sectional and longitudinal analyses. The cross-sectional analyses involved 440,645 participants (54.0% female; mean [SD] age, 56.5 [8.1] years), among whom 14,105 (3.2%) were identified with cataract. The longitudinal analyses included 426,540 participants (53.9% female; mean [SD] age, 56.3 [8.1] years). Over a mean follow-up period of 10.8 years, 55,658 incident cataract cases were documented. Shared characteristics were noted among participants with cataract in both the cross-sectional and longitudinal analyses. These individuals tended to be older, female, have lower educational attainment, be former smokers, non-drinkers, have higher BMI and sun exposure, lower sleep quality, longer sedentary time, and engage in lower levels of PA. Furthermore, individuals with cataract showed a higher likelihood of having a medical history of diabetes or hypertension.

**Table 1:** Baseline characteristics of study participants by cataract status

**Characteristics **^**a**^	**Cross-sectional analyses**			**Longitudinal analyses**		
	**Non-cataract** **(N = 426,540)**	**Cataract** **(N = 14,105)**	*P* value	**Non-cataract** **(N = 370,882)**	**Cataract** **(N = 55,658)**	*P* value ^b^
Age (years)	56.3 (8.1)	62.3 (6.0)	< 0.001	55.5 (8.1)	61.7 (5.9)	< 0.001
Sex			< 0.001			< 0.001
Male	196,782 (46.1)	5910 (41.9)		173,313 (46.7)	23,469 (42.2)	
Female	229,758 (53.9)	8195 (58.1)		197,569 (53.3)	32,189 (57.8)	
Ethnicity			< 0.001			< 0.001
Whites	404,427 (94.8)	12,852 (91.1)		351,895 (94.9)	52,532 (94.4)	
Non-whites	21,958 (5.1)	1243 (8.8)		18,849 (5.1)	3109 (5.6)	
Education level			< 0.001			< 0.001
NVQ/CSE/A levels/others	211,663 (49.6)	6664 (47.2)		185,419 (50.0)	26,244 (47.2)	
College or university degree	147,348 (34.5)	4430 (31.4)		130,567 (35.2)	16,781 (30.2)	
None	64,223 (15.1)	2872 (20.4)		52,149 (14.1)	12,074 (21.7)	
Townsend deprivation index	−1.4 (3.0)	−1.2 (3.1)	< 0.001	−1.4 (3.0)	−1.4 (3.1)	0.014
Smoking status			< 0.001			< 0.001
Never	233,160 (54.7)	7397 (52.4)		205,045 (55.3)	28,115 (50.5)	
Former	145,516 (34.1)	5424 (38.5)		123,732 (33.4)	21,784 (39.1)	
Current	41,455 (9.7)	1080 (7.7)		36,993 (10.0)	4462 (8.0)	
Drinking status			< 0.001			< 0.001
Never	16,943 (4.0)	879 (6.2)		14,128 (3.8)	2815 (5.1)	
Former	14,114 (3.3)	653 (4.6)		12,059 (3.3)	2055 (3.7)	
Current	395,167 (92.6)	12,560 (89.0)		344,429 (92.9)	50,738 (91.2)	
Sun exposure (h/day)	3.7 (2.4)	3.9 (2.3)	0.001	3.7 (2.4)	3.9 (2.3)	< 0.001
Diabetes	37,951 (8.9)	2466 (17.5)	< 0.001	29,281 (7.9)	8670 (15.6)	< 0.001
Hypertension	165,746 (39.0)	7790 (55.4)	< 0.001	135,334 (36.5)	30,412 (54.6)	< 0.001
BMI (kg/m^2^)	27.26 (4.6)	27.66 (4.8)	< 0.001	27.21 (4.7)	27.54 (4.7)	< 0.001
Sleep scores	3.5 (1.0)	3.4 (1.1)	< 0.001	3.6 (1.0)	3.5 (1.1)	< 0.001
Sleep pattern						
Poor	12,971 (3.0)	598 (4.2)	< 0.001	11,012 (3.0)	1959 (3.5)	< 0.001
Intermediate	181,225 (42.5)	6482 (46.0)		156,484 (42.2)	24,741 (44.5)	
Healthy	232,344 (54.5)	7025 (49.8)		203,386 (54.8)	28,958 (52.0)	
PA (MET-mins/week)	2577.6 (2737.8)	2482.1 (2669.2)	0.001	2583.9 (2748.7)	2535.5 (2663.7)	< 0.001
PA group			< 0.001			< 0.001
Low	83,776 (19.6)	2941 (20.9)		72,583 (19.6)	11,193 (20.1)	
Moderate	79,913 (18.7)	2730 (19.4)		69,240 (18.7)	10,673 (19.2)	
High	262,851 (61.6)	8434 (59.8)		229,059 (61.8)	33,792 (60.7)	
SB (h/day)	4.5 (2.4)	4.5 (2.4)	0.109	4.4 (2.4)	4.5 (2.3)	< 0.001
SB group			< 0.001			< 0.001
Low	160,078 (37.5)	4962 (35.2)		140,702 (37.9)	19,376 (34.8)	
Moderate	148,681 (34.9)	4998 (35.4)		128,518 (34.7)	20,163 (36.2)	
High	117,781 (27.6)	4145 (29.4)		101,662 (27.4)	16,119 (29.0)	

*Abbreviations:* *BMI* Body mass index, *PA* Physical activity, *MET* Metabolic equivalent of task, *SB* Sedentary behavior

^a^Shown are numbers (%) or means (standard deviations)

^b^*P*-values are derived using either Student’s t-test or Chi-square test

#### Independent association of exposures with cataract in cross-sectional analyses

After adjustments for age and sex, individuals with poor sleep demonstrated a 51% rise in the odds of cataract (OR, 1.51; 95% CI, 1.38–1.65). Moreover, even after further adjustments for all variables in Model 2, this relationship retained statistical significance, with an OR of 1.35 (95% CI, 1.23–1.48). Similarly, individuals with moderate and low PA levels exhibited a notable correlation with cataract in contrast to individuals with high PA levels, following adjustments for age and sex (Table 2). Nevertheless, upon complete adjustments for all variables in Model 2, solely low PA levels were found to have a significant association with cataract, with an OR of 1.06 (95% CI, 1.01–1.11). The high SB group exhibited a nearly significant association (OR, 1.05; 95% CI, 1.00–1.10; *P* = 0.065) when compared to the low SB group in Model 2.

**Table 2:** The cross-sectional and longitudinal associations of sleep scores, sedentary behavior, and physical activity with cataract

**Exposure **^**a**^	**Cross-sectional associations**		**Longitudinal associations**	
	**OR (95% CI)**		**HR (95% CI)**	
	**Model 1 **^**b**^	**Model 2 **^**c**^	**Model 1 **^**b**^	**Model 2 **^**c**^
**Sleep pattern**				
Poor	1.51 (1.38 to 1.65)^***^	1.35 (1.23 to 1.48)^***^	1.27 (1.21 to 1.32)^***^	1.14 (1.08 to 1.19)^***^
Intermediate	1.16 (1.12 to 1.20)^***^	1.11 (1.07 to 1.16)^***^	1.09 (1.08 to 1.11)^***^	1.05 (1.04 to 1.07)^***^
Healthy	1.00	1.00	1.00	1.00
**Sedentary behavior**				
High	1.07 (1.03 to 1.12)^**^	1.05 (1.00 to 1.10)	1.13 (1.11 to 1.15)^***^	1.08 (1.06 to 1.11)^**^
Moderate	0.97 (0.93 to 1.01)	0.99 (0.94 to 1.02)	1.05 (1.02 to 1.07)^***^	1.03 (1.01 to 1.05)^***^
Low	1.00	1.00	1.00	1.00
**Physical activity**				
Low	1.15 (1.11 to 1.20)^***^	1.06 (1.01 to 1.11)^*^	1.11 (1.08 to 1.13)^***^	1.05 (1.02 to 1.07)^***^
Moderate	1.09 (1.04 to 1.14)^***^	1.04 (0.99 to 1.09)	1.07 (1.05 to 1.09)^***^	1.05 (1.02 to 1.07)^***^
High	1.00	1.00	1.00	1.00

*Abbreviations:* *OR* Odds ratio, *HR* Hazard ratio, *CI* Confidence interval, *MET* Metabolic equivalent task

^a^Sleep scores were categorized into: poor, 0–1; intermediate, 2–3; healthy, 4–5. Sedentary behavior was categorized into: low, < 4 h/day; moderate, 4–5 h/day; high, ≥ 6 h/day. Physical activity levels were categorized based on public health guidelines: low (< 600 MET-mins/week); moderate (600 to < 1200 MET-mins/week); and high (≥ 1200 MET-mins/week)

^b^Adjusted for age and sex

^c^Further adjusted for ethnicity, socioeconomic status, education, body mass index, smoking status, alcohol consumption, sun exposure, diabetes and hypertension

^*^P < 0.05, ^**^P < 0.01, ^***^P < 0.001

#### Joint association of exposures with cataract in cross-sectional analyses

In terms of the combined association of PA and SB, individuals characterized by low PA and high SB demonstrated a notable correlation with cataract, compared to individuals with high PA and low SB (OR, 1.14; 95% CI, 1.05–1.23; Additional file 2: Table S4). Individuals with intermediate or poor sleep patterns displayed increased odds of cataract when exposed to a combination of moderate to high SB or any levels of PA (Additional file 2: Tables S5-6). When estimating the combined associations of sleep, SB, and PA, each exposure was re-categorized into binary variables, leading to the formation of eight combination subgroups (Table 3). Within the various combinations, individuals characterized by poor sleep quality, high SB, and engagement in non-ideal PA demonstrated the highest odds of cataract (OR, 1.73; 95% CI, 1.37–2.15), in comparison to the reference group characterized by good sleep, low to moderate SB levels, and participation in ideal PA.

**Table 3:** The joint associations of sleep scores, sedentary behavior, and physical activity with cataract

Exposure ^a^	Cross-sectional associations		Longitudinal associations	
	**OR (95% CI) **^**b**^	*P* value	**HR (95% CI) **^**b**^	*P* value
**Low to moderate SB**				
Good sleep & ideal PA	1.00	-	1.00	-
Good sleep & non-ideal PA	1.13 (0.98 to 1.30)	0.095	1.02 (0.99 to 1.05)	0.123
Poor sleep & ideal PA	1.01 (0.95 to 1.07)	0.838	1.08 (1.01 to 1.16)	0.028
Poor sleep & non-ideal PA	1.47 (1.18 to 1.81)	< 0.001	1.08 (0.95 to 1.22)	0.263
**High SB**				
Good sleep & ideal PA	1.03 (0.98 to 1.19)	0.243	1.06 (1.03 to 1.08)	< 0.001
Good sleep & non-ideal PA	1.10 (1.02 to 1.19	0.009	1.09 (1.05 to 1.13)	< 0.001
Poor sleep & ideal PA	1.30 (1.08 to 1.55)	0.004	1.15 (1.04 to 1.26)	0.004
Poor sleep & non-ideal PA	1.73 (1.37 to 2.15)	< 0.001	1.37 (1.21 to 1.55)	< 0.001

*Abbreviations:* *OR* Odds ratio, *HR* Hazard ratio, *CI* Confidence interval, *MET* Metabolic equivalent task

^a^Sleep scores were categorized into: poor, 0–1; good, 2–5. Sedentary behavior was categorized into: low to moderate, < 6 h/day; high, ≥ 6 h/day. Physical activity levels were categorized into: non-ideal physical activity (< 600 MET-mins/week); ideal physical activity (≥ 600 MET-mins/week)

^b^Adjusted for age, sex, ethnicity, socioeconomic status, education, body mass index, smoking status, alcohol consumption, sun exposure, diabetes and hypertension

#### Independent association of exposures with cataract in longitudinal analyses

**Fig. 1:**
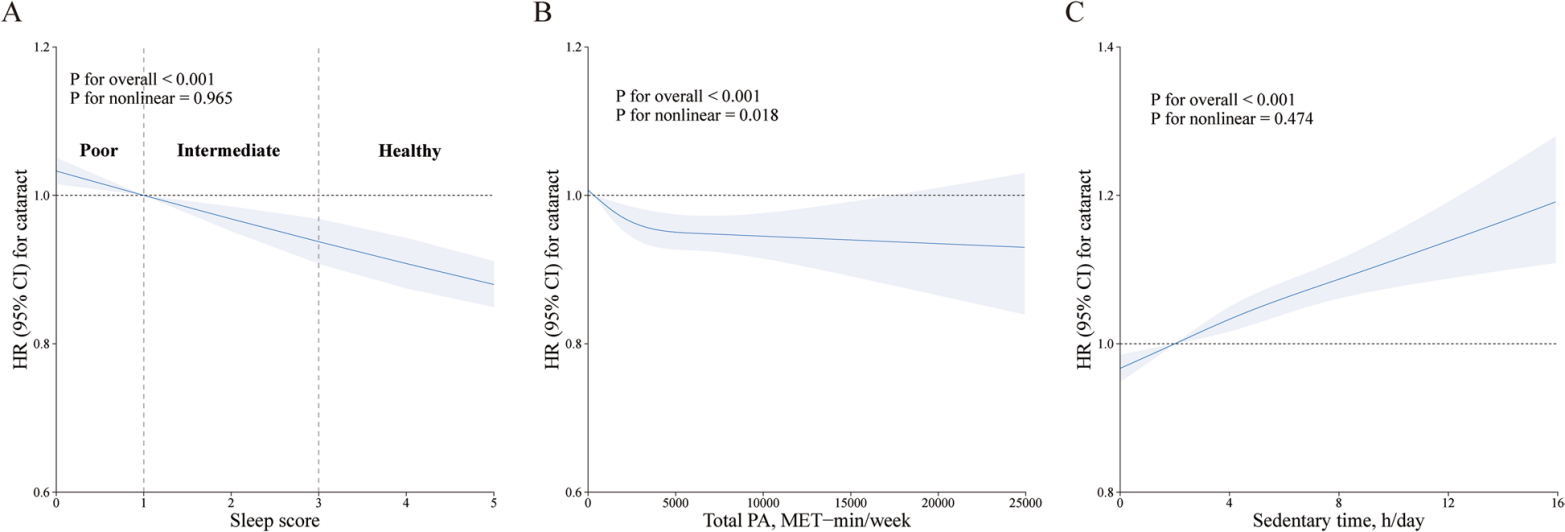
The dose–response association of sleep score, total PA, sedentary time with the risk of cataract. Dose–response associations between sleep score (A), total PA (B), and sedentary time (C). Restricted cubic splines were constructed with three knots located at the 10th, 50th and 90th percentiles of each exposure. The models were adjusted for age, sex, ethnicity, socioeconomic status, education, body mass index, smoking status, alcohol status, sun exposure, diabetes and hypertension. HR, hazard ratio; CI, confidence interval; PA, physical activity

When compared to the healthy sleep group, the hazard ratios (HRs) for developing cataract were elevated in both the intermediate group (HR, 1.09; 95% CI, 1.08–1.11) and the poor sleep group (HR, 1.27; 95% CI, 1.21–1.32) after adjusting for age and sex. After full adjustments in Model 2, this association remained consistent (Table 2). Furthermore, a linear inverse relationship was observed between sleep score and cataract risk (*P* for nonlinearity = 0.965; Fig. 1). In contrast to the low SB group, both the moderate and high SB groups demonstrated an increased likelihood of cataract development, with HRs of 1.03 (95% CI, 1.01–1.05) and 1.08 (95% CI, 1.06–1.11), respectively. Similarly, a dose–response relationship was found between sedentary time and cataract risk (*P* for nonlinearity = 0.474, Fig. 1). In parallel, moderate and low levels of PA were associated with a 5% rise in the risk of developing cataract after accounting for all variables in Model 2 (Table 2). Total PA demonstrated an L-shaped association with cataract incidence, with the lowest risk observed at approximately 5000 MET-min/week of PA (Fig. 1).

In a subsample of 91,127 participants with valid accelerometer-derived data, both light-intensity physical activity (LIPA) and moderate-to-vigorous-intensity physical activity (MVPA) exhibited L-shaped associations with cataract incidence. The lowest risk was observed at approximately 5 h/day for LIPA and 500 min/week for MVPA (Additional file 1: Fig. S3). A dose–response relationship was identified between sedentary time and cataract risk (*P* for nonlinearity = 0.065; Additional file 1: Fig. S3). However, no significant association was observed between sleep duration and cataract incidence (Additional file 1: Fig. S3).

#### Joint association of exposures with cataract in longitudinal analyses

Individuals who had a combination of high SB and any levels of PA showed an increased risk of cataract development compared to those with low SB and high PA (Additional file 2: Table S4). Among individuals with poor sleep, the combination of any levels of PA or SB was notably associated with an increased risk of cataract development (Additional file 2: Tables S5-6). In comparison to the reference group characterized by good sleep, low to moderate SB, and ideal PA, participants with a combination of poor sleep, high SB, and non-ideal PA had a 37% increased risk of developing cataract (HR, 1.37; 95% CI, 1.21–1.55). Furthermore, in joint analyses, sleep and PA demonstrated significant associations with cataract on both additive and multiplicative scales (HR, 1.03, 95% CI 1.00 to1.07). Meanwhile, SB and PA exhibited additive interaction, and sleep and SB showed multiplicative interaction in relation to cataract (Additional file 2: Tables S7-9).

### Isotemporal substitution models

**Fig. 2:**
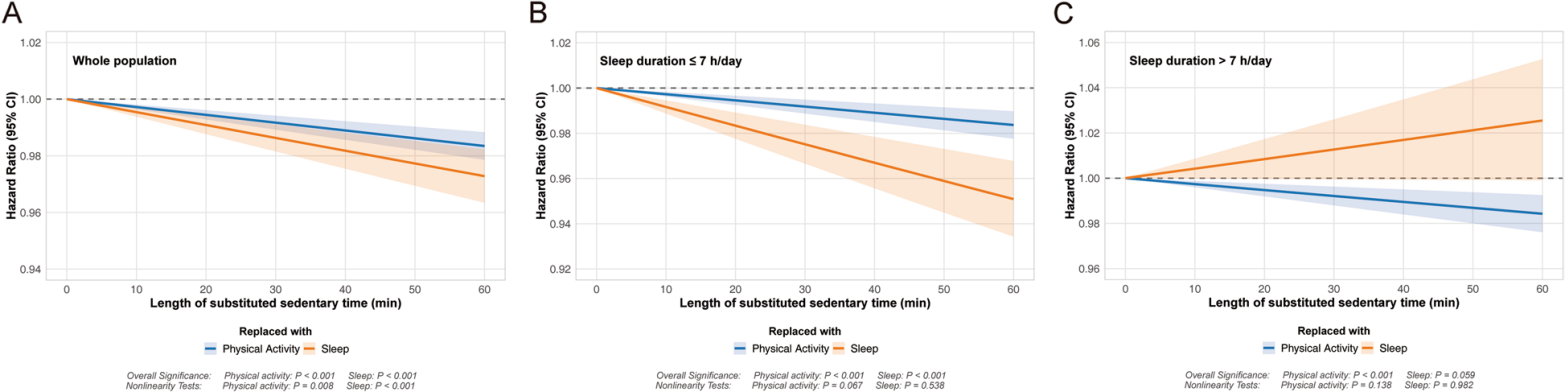
Hazard ratios for substituting sedentary time with physical activity or sleep (A) in whole population; (B) in participants with sleep duration ≤ 7 h/day and (C) > 7 h/day. The models were adjusted for age, sex, ethnicity, socioeconomic status, education, body mass index, smoking status, alcohol consumption, sun exposure, diabetes and hypertension. CI, confidence interval

In the overall population, replacing 1 h/day of SB with PA (HR, 0.983; 95% CI, 0.978–0.988) or sleep (HR, 0.973; 95% CI, 0.963–0.982) was associated with a reduced risk of incident cataract (Table 4). Given that 7 h/day of questionnaire-derived sleep duration corresponded to the lowest cataract risk (Additional file 1: Fig. S4), we further investigated the substitution effects stratified by sleep duration (≤ 7 h/day vs. > 7 h/day). As showed in Table 4 and Fig. 2, among individuals sleeping ≤ 7 h/day, replacing 1 h of SB with equivalent PA was associated with a 1.6% reduction in cataract risk (HR, 0.984; 95% CI, 0.978–0.990), while substitution with sleep reduced cataract risk by 4.9% (HR, 0.951; 95% CI, 0.934–0.968). Conversely, for those sleeping > 7 h/day, replacing SB with PA remained significantly associated with a 1.6% of risk reduction (HR, 0.984; 95% CI, 0.976–0.992), whereas substitution with sleep demonstrated no significant association (HR, 1.025; 95% CI, 0.999–1.053).

**Table 4:** Associations of sleep and physical activity with incident cataract by replacing 1 h of sedentary time using isotemporal substitution model

**Activity**	**Model 1**^a^		**Model 2**^b^	
	**HR (95% CI)**	*P* value	**HR (95% CI)**	*P* value
**Whole population**				
PA	0.971 (0.967–0.976)	< 0.001	0.983 (0.978–0.988)	< 0.001
Sleep	0.956 (0.948–0.965)	< 0.001	0.973 (0.963–0.982)	< 0.001
**Sleep duration ≤ 7 h/day**				
PA	0.975 (0.969–0.980)	< 0.001	0.984 (0.978–0.990)	< 0.001
Sleep	0.917 (0.902–0.932)	< 0.001	0.951 (0.934–0.968)	< 0.001
**Sleep duration > 7 h/day**				
PA	0.969 (0.962–0.977)	< 0.001	0.984 (0.976–0.992)	< 0.001
Sleep	1.047 (1.022–1.073)	< 0.001	1.025 (0.999–1.053)	0.059

*Abbreviations:* *HR* Hazard ratio, *CI* Confidence interval, *PA* Physical activity

^a^Adjusted for age and sex

^b^Adjusted for age, sex, ethnicity, socioeconomic status, education, body mass index, smoking status, alcohol consumption, sun exposure, diabetes and hypertension

Accelerometer-derived data further demonstrated that substituting 1 h of SB with equivalent durations of LIPA (HR, 0.980; 95% CI, 0.965–0.995), MVPA (HR, 0.943; 95% CI, 0.903–0.985), or sleep (HR, 0.982; 95% CI, 0.964–1.000) was associated with reduced incident cataract risk in the overall population (Additional file 2: Table S10). When stratified by sleep duration, replacing SB with LIPA significantly reduced cataract risk among individuals sleeping ≤ 7 h/day. Conversely, in those sleeping > 7 h/day, only MVPA substitution was associated with a reduced risk of incident cataract (Additional file 1: Fig. S5; Additional file 2: Table S10).

### Subgroup and sensitivity analyses

When the analyses were stratified by age, sex, and diabetes, the overall findings of the study remained consistent (Additional file 2: Tables S11-13). Notably, the impact of sleep and SB on cataract development was significantly more pronounced during midlife (P for interaction < 0.05). Additionally, the influence of PA on cataract development was significantly more prominent in males (P for interaction < 0.05).

The sensitivity analysis consistently revealed independent associations between each exposure and cataract development, even after adjusting for the mutual adjustments of PA and SB in sleep pattern, sleep and PA in SB, or sleep and SB in PA, along with covariates in Model 2 (Additional file 2: Table S14). In addition, after further adjusting for eye trauma, myopia, and corticosteroid use, the results were still consistent (Additional file 2: Table S14). Moreover, after excluding individuals with less than 2 years of follow-up, the findings remained stable (Additional file 2: Table S15).

## Discussion

The results of our study demonstrate that sleep, PA and SB each independently contribute to the risk of cataract development. Moreover, when these variables are considered together, they exhibit even greater risks for cataract development, suggesting a potential synergistic effect among these movement behaviors. Additionally, the combination of poor sleep, high SB and low levels of PA indicated the highest risk of cataract in both the cross-sectional and prospective analysis. Replacing sedentary time with equivalent PA or sleep could reduce the risk of incident cataract.

Existing evidence regarding the relationship between sleep and cataract development remains sparse. To our knowledge, this study is the first to use a composite healthy sleep score, integrating five distinct sleep characteristics, to investigate the relationship between sleep and cataract risk. Our analyses revealed significant associations between poor sleep and elevated cataract risk in both cross-sectional and longitudinal analyses. While prior work identified sleep apnea as a risk factor for cataract progression [15], conflicting evidence exists, with another study reporting no significant association between sleep disruption and cataract incidence [16]. Notably, accelerometer-derived sleep duration alone showed no correlation with cataract risk (Additional file 1: Fig. S3), underscoring the discrepancy between isolated sleep metrics and composite sleep quality. These inconsistencies highlight the complexity of the role of sleep in cataractogenesis and emphasize the necessity of prioritizing holistic sleep assessments over singular dimensions such as duration. Importantly, even among individuals with high PA levels, poor sleep persisted as an independent risk factor for cataract, reinforcing the dominant influence of sleep on cataract. The potential mechanism between sleep and cataractogenesis may include: 1) impaired antioxidant defenses due to sleep disturbances, exacerbating oxidative stress within the lens [39]; 2) circadian rhythm dysregulation, disrupting metabolic homeostasis and glutathione-dependent repair mechanisms in the lens [40]; and 3) systemic inflammation driven by elevated pro-inflammatory cytokines, such as C-reactive protein and interleukin-6, which may permeate ocular tissues and promote crystallin protein aggregation, a hallmark of cataract formation [41].

The findings in our study are consistent with previous studies [17, 18, 20, 42], supporting the associations between low levels of PA and an elevated risk of cataract, using both questionnaire and accelerometer measured data. In contrast, a study from the Beijing Eye Study found no significant association between PA and cataract prevalence [19], possibly due to differences in PA assessment methods and limited sample size. Furthermore, SB, increasingly prevalent in modern lifestyles, has been linked to various health outcomes [43–46]. Similar to the results of Wang et al. [19], our study did not find a significant cross-sectional association between SB and cataract. However, in prospective analysis, our findings indicated that sedentary time were linearly associated with cataract risk by using questionnaire and accelerometer derived data. Moreover, our study suggests that the detrimental impact of high SB on cataract development may be more pronounced in individuals with lower PA levels or poorer sleep patterns (Additional file 2: Tables S4-5).

Emerging evidence has highlighted potential synergistic effects of sleep, PA, and SB on diverse health outcomes [47–50]. However, their combined impact on cataract pathogenesis has not been investigated. In this study, we have identified multiplicative or additive interactions among these three movement-related behaviors. Specifically, any combination of (1) poor sleep and high SB, (2) poor sleep and low PA, (3) high SB and low PA, or (4) poor sleep, high SB, and low PA demonstrated greater adverse effects on cataract risk than their individual contributions. These findings suggest that interventions targeting isolated behaviors may fail to account for interconnected biological and behavioral cascades. For instance, increasing PA may concurrently reduce SB and enhance sleep quality, establishing a synergistic protective feedback loop [51, 52]. Additionally, reducing screen time has been associated with improved sleep regulation [53–55]. Furthermore, isotemporal substitution modeling revealed differential benefits: for individuals sleeping ≤ 7 h/day, replacing SB with sleep yielded greater reductions in cataract risk. Conversely, for those sleeping > 7 h/day, substituting SB with PA emerged as the optimal strategy. Consequently, public health strategies should prioritize integrated interventions addressing sleep, PA, and SB collectively to maximize synergistic protective effects.

The strength of this study is that it was based on the UK Biobank, a prospective cohort study with a substantial sample size, long-term follow-up, and meticulous evaluation of confounding variables. These elements facilitated a comprehensive exploration of the relationships between the investigated exposures and cataract risk. However, it is important to acknowledge the potential limitations of our study. Firstly, the data about sleep, PA, and SB relied on self-reported information, which may introduce reporting bias and impact result validity. However, we repeated the main analysis in a subsample using accelerometer-derived data to examine the prospective relationship between exposures and cataract. The findings consistently highlighted the role of these movement behaviors in cataract development. Secondly, all exposures and confounders were evaluated at baseline, assuming their stability throughout the follow-up period. Nonetheless, future research delving into the temporal dynamics of these behaviors would provide valuable insights into their combined effects. Thirdly, although we adopted the composite sleep score, this system has not undergone formal validation. However, its individual components, such as sleep duration, insomnia, and snoring, have been validated by the UK Biobank and this system were widely applied in numerous previous UK Biobank studies [25, 26, 56, 57]. Fourthly, cataract ascertainment in UK Biobank relies primarily on hospital records and some cataract cases might have been missed. Our observed incidence rate (1,208 per 100,000 person-years) is notably lower than the 2024 UK national cataract surgery rate (5,160 per 100,000 per year) [58]. While surgical rates provide a conservative proxy for disease incidence, this suggests our cohort’s incidence is lower than expected in the general UK population. Therefore, the observed associations between these behaviors and cataract risk may be attenuated. Fifthly, despite careful adjustments for potential confounders, residual confounding factors may persist due to the inherent constraints of an observational study. Lastly, caution should be exercised when generalizing these findings to other racial or age demographics, given that the predominant participants in our study were middle-aged or elderly individuals of White ethnicity.

## Conclusions

In conclusion, our study demonstrates the associations between suboptimal sleep, high SB, low PA, and elevated cataract risk. Replacing SB with PA or adequate sleep could attenuate cataract risk. Targeting ≥ 600 MET-min/week of PA, limiting SB to < 6 h/day, obtaining 7–8 h of nightly sleep, and improving sleep quality are likely to yield the greatest reduction in cataract risk. These findings underscore the importance of modifying these daily behaviors, either individually or in combination, to effectively mitigate the risk of cataract development.

## Supplementary Information

Additional file 1: Figures S1- S5. Fig. S1. Participant flow diagram for questionnaire-derived movement behaviors analyses. Fig. S2. Participant flow diagram for accelerometer-derived movement behaviors analyses. Fig. S3. The dose-response association of accelerometer-derived movement behaviors with the risk of cataract. Fig. S4. The dose-response association of questionnaire-derived sleep duration with the risk of cataract. Fig. S5. Hazard ratios for substituting accelerometer-derived sedentary time with LIPA, MVPA or sleep.

Additional file 2: Supplementary methods, Tables S1-S15. Table S1. Variable ID and Codes Used for Cataract Diagnosis in the UK Biobank. Table S2. The scoring system of sleep. Table S3. The resource and definition of the selected covariates (except for age, and sex). Table S4. The joint associations of sedentary behavior, and physical activity with cataract. Table S5. The joint associations of sedentary behavior, and sleep scores with cataract. Table S6. The joint associations of sleep scores and physical activity with cataract. Table S7. Analyses on interaction of sleep scores and physical activity with incident cataract. Table S8. Analyses on interaction of sedentary behavior and physical activity with incident cataract. Table S9. Analyses on interaction of sleep scores and sedentary behavior with incident cataract. Table S10. Associations of accelerometer-derived sleep and physical activity with incident cataract by replacing sedentary behavior using isotemporal substitution model. Table S11. The longitudinal associations of sleep scores, sedentary behavior, and physical activity with cataract stratified by age. Table S12. The longitudinal associations of sleep scores, sedentary behavior, and physical activity with cataract stratified by sex. Table S13. The longitudinal associations of sleep scores, sedentary behavior, and physical activity with cataract stratified by diabetes. Table S14. The longitudinal associations between sleep scores, sedentary behavior, and physical activity and cataract with additional adjustment. Table S15. The associations of sleep scores, sedentary behavior, and physical activity with cataract after excluding participants with less than 2 years of follow-up.

## Data Availability

All UK Biobank information is available online (https://www.ukbiobank.ac.uk/). Data access is available through applications. This research has been conducted using the UK Biobank Resource under Application Number 91320.

## Abbreviations

PA: Physical activity
SB: Sedentary behavior
OR: Odds ratio
HR: Hazard ratio
CI: Confidence interval
MET: Metabolic equivalents
BMI: Body mass index
ICD: International Classification of Diseases
OPCS: Classification of Surgical Operations and Procedures
WHO: World Health Organization
LIPA: Light intensity of physical activity
MVPA: Moderate-to-vigorous intensity of physical activity
IPAQ: International Physical Activity Questionnaire
