# Supplementary material for: Sleep, physical activity, sedentary behavior, and risk of cataract: a cross-sectional and prospective study from UK Biobank
Source: BMC Med. 2025 Aug 8;23:466. doi: 10.1186/s12916-025-04312-7 (PMC12333188; doi:10.1186/s12916-025-04312-7)
Supplement: Supplementary file 1 — Additional file 1: Figures S1- S5. Fig. S1. Participant flow diagram for questionnaire-derived movement behaviors analyses. Fig. S2. Participant flow diagram for accelerometer-derived movement behaviors analyses. Fig. S3. The dose-response association of accelerometer-derived movement behaviors with the risk of cataract. Fig. S4. The dose-response association of questionnaire-derived sleep duration with the risk of cataract. Fig. S5. Hazard ratios for substituting accelerometer-derived sedentary time with LIPA, MVPA or sleep. [file 12916_2025_4312_MOESM1_ESM.pdf]

Additional File 1: Figures S1 – S5.

Fig. S1. Participant flow diagram for questionnaire-derived movement behaviors analyses.

Fig. S2. Participant flow diagram for accelerometer-derived movement behaviors analyses.

Fig. S3. The dose-response association of accelerometer-derived movement behaviors with the risk of cataract.

Fig. S4. The dose-response association of questionnaire-derived sleep duration with the risk of cataract.

Fig. S5. Hazard ratios for substituting accelerometer-derived sedentary time with LIPA, MVPA or sleep.

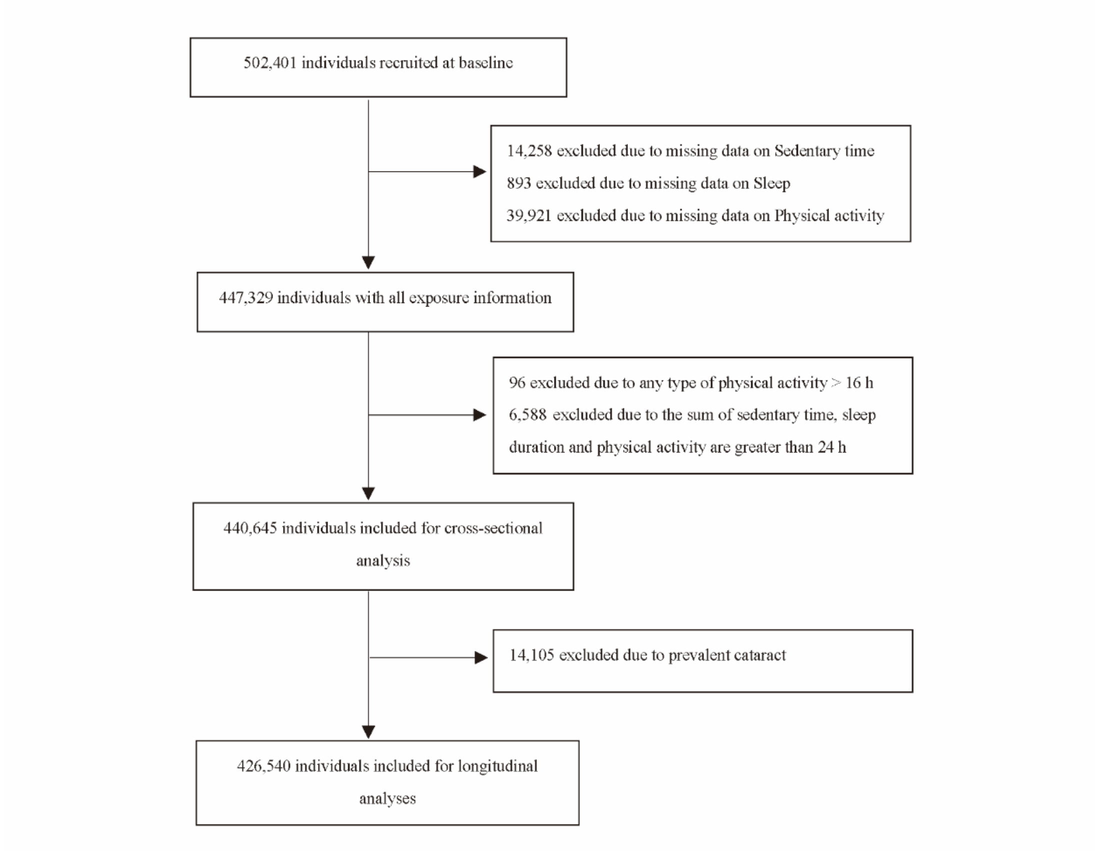

Fig. S1. Participant flow diagram for questionnaire-derived movement behaviors analyses.

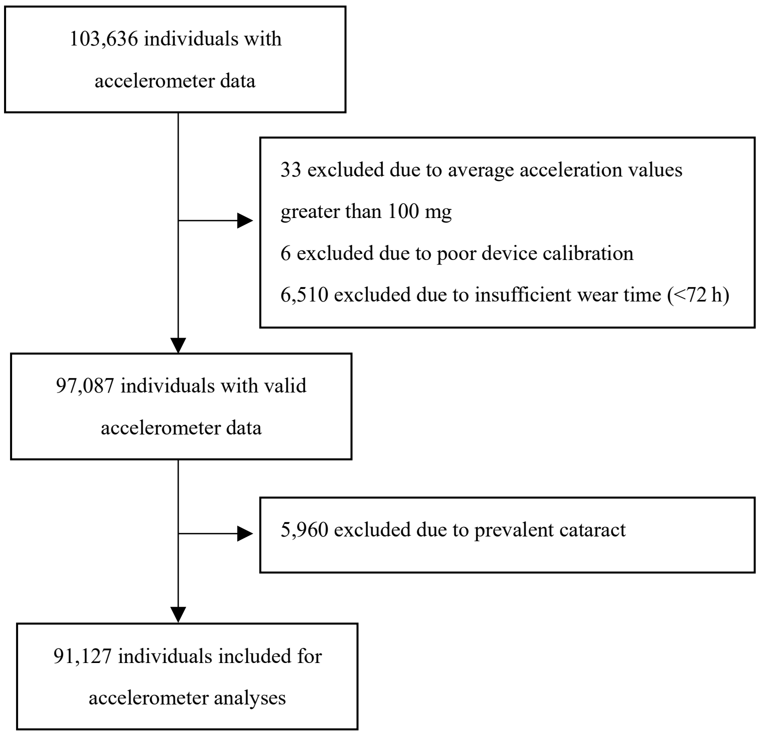

Fig. S2. Participant flow diagram for accelerometer-derived movement behaviors analyses.

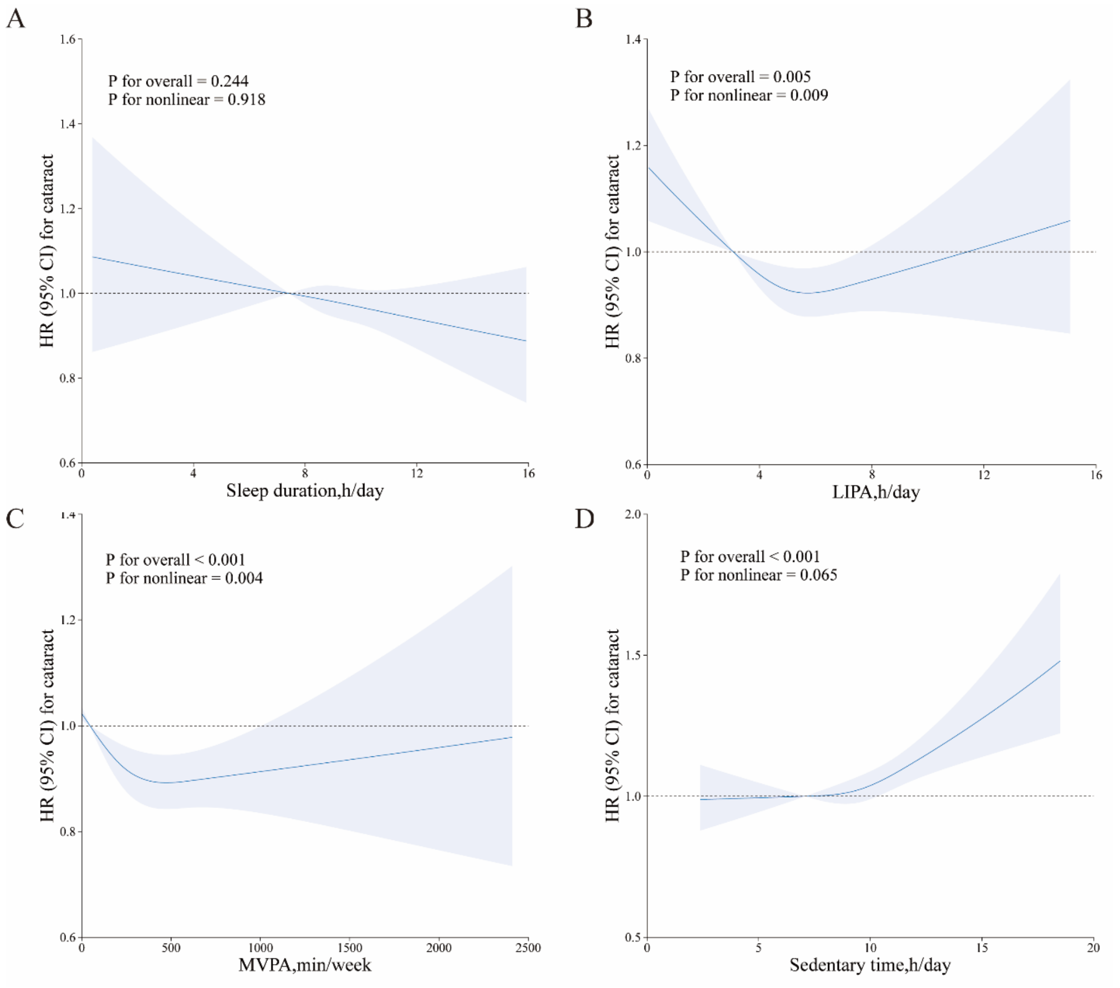

Fig. S3. The dose-response association of accelerometer-derived movement behaviors with the risk of cataract. Dose-response associations between accelerometer-measured sleep duration (A), LIPA (B), MVPA (C) and sedentary time (D). Restricted cubic splines were constructed with three knots located at the 10th, 50th and 90th percentiles of each exposure. The models were adjusted for age, sex, ethnicity, socioeconomic status, education, body mass index, smoking status, alcohol consumption, sun exposure, diabetes and hypertension. HR, hazard ratio; CI, confidence interval; MVPA, moderate-to-vigorous physical activity; LIPA, light intensity of physical activity.

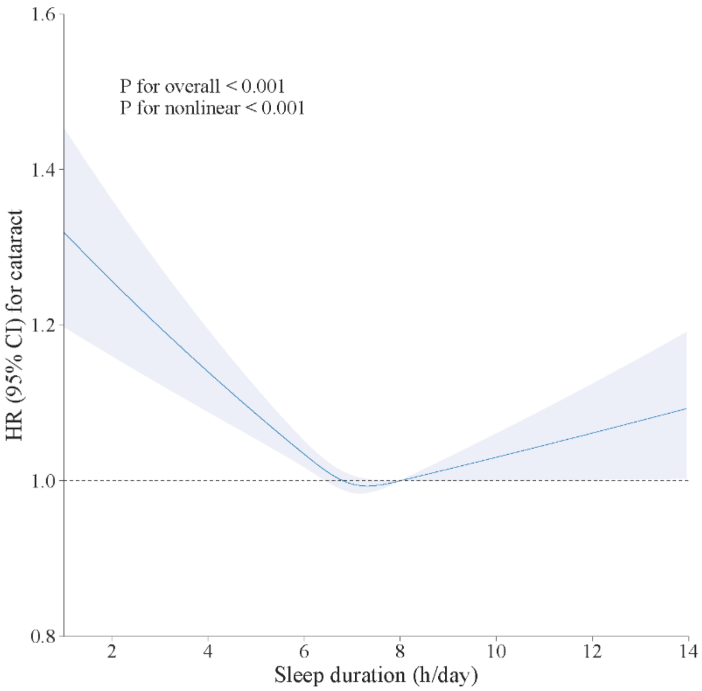

Fig. S4. The dose-response association of questionnaire-derived sleep duration with the risk of cataract. Restricted cubic splines were constructed with three knots located at the 10th, 50th and 90th percentiles. The models were adjusted for age, sex, ethnicity, socioeconomic status, education, body mass index, smoking status, alcohol consumption, sun exposure, diabetes and hypertension. HR, hazard ratio; CI, confidence interval.

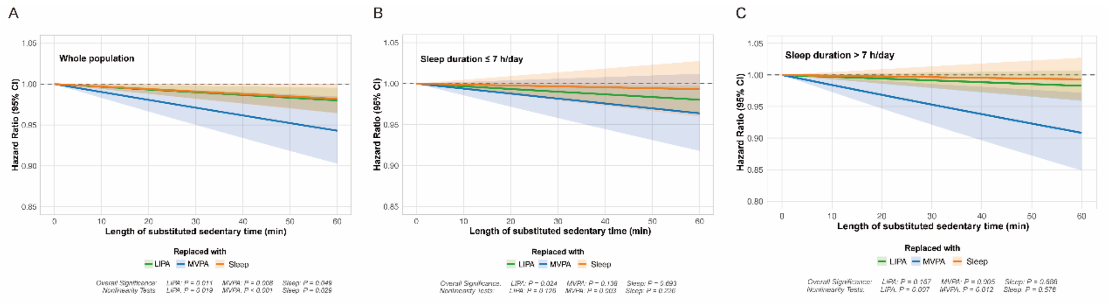

Fig. S5. Hazard ratios for substituting accelerometer-derived sedentary time with LIPA, MVPA or sleep (A) in whole population; (B) in participants with sleep duration ≤7 h/day and (C) >7 h/day. The models were adjusted for age, sex, ethnicity, socioeconomic status, education, body mass index, smoking status, alcohol consumption, sun exposure, diabetes and hypertension. CI, confidence interval; MVPA, moderate-to-vigorous physical activity; LIPA, light intensity of physical activity.
