# Supplementary material for: Sleep, physical activity, sedentary behavior, and risk of cataract: a cross-sectional and prospective study from UK Biobank
Source: BMC Med. 2025 Aug 8;23:466. doi: 10.1186/s12916-025-04312-7 (PMC12333188; doi:10.1186/s12916-025-04312-7)
Supplement: Supplementary file 2 — Additional file 2: Supplementary methods, Tables S1-S15. Table S1. Variable ID and Codes Used for Cataract Diagnosis in the UK Biobank. Table S2. The scoring system of sleep. Table S3. The resource and definition of the selected covariates (except for age, and sex). Table S4. The joint associations of sedentary behavior, and physical activity with cataract. Table S5. The joint associations of sedentary behavior, and sleep scores with cataract. Table S6. The joint associations of sleep scores and physical activity with cataract. Table S7. Analyses on interaction of sleep scores and physical activity with incident cataract. Table S8. Analyses on interaction of sedentary behavior and physical activity with incident cataract. Table S9. Analyses on interaction of sleep scores and sedentary behavior with incident cataract. Table S10. Associations of accelerometer-derived sleep and physical activity with incident cataract by replacing sedentary behavior using isotemporal substitution model. Table S11. The longitudinal associations of sleep scores, sedentary behavior, and physical activity with cataract stratified by age. Table S12. The longitudinal associations of sleep scores, sedentary behavior, and physical activity with cataract stratified by sex. Table S13. The longitudinal associations of sleep scores, sedentary behavior, and physical activity with cataract stratified by diabetes. Table S14. The longitudinal associations between sleep scores, sedentary behavior, and physical activity and cataract with additional adjustment. Table S15. The associations of sleep scores, sedentary behavior, and physical activity with cataract after excluding participants with less than 2 years of follow-up. [file 12916_2025_4312_MOESM2_ESM.pdf]

Additional file 2: Supplementary methods, Tables S1-S15

Supplementary methods

Table S1. Variable ID and Codes Used for Cataract Diagnosis in the UK Biobank.

Table S2. The scoring system of sleep.

Table S3. The resource and definition of the selected covariates (except for age, and sex).

Table S4. The joint associations of sedentary behavior, and physical activity with cataract.

Table S5. The joint associations of sedentary behavior, and sleep scores with cataract.

Table S6. The joint associations of sleep scores and physical activity with cataract.

Table S7. Analyses on interaction of sleep scores and physical activity with incident cataract.

Table S8. Analyses on interaction of sedentary behavior and physical activity with incident cataract.

Table S9. Analyses on interaction of sleep scores and sedentary behavior with incident cataract.

Table S10. Associations of accelerometer-derived sleep and physical activity with incident cataract by replacing sedentary behavior using isotemporal substitution model.

Table S11. The longitudinal associations of sleep scores, sedentary behavior, and physical activity with cataract stratified by age.

Table S12. The longitudinal associations of sleep scores, sedentary behavior, and physical activity with cataract stratified by sex.

Table S13. The longitudinal associations of sleep scores, sedentary behavior, and physical activity with cataract stratified by diabetes.

Table S14. The longitudinal associations between sleep scores, sedentary behavior, and physical activity and cataract with additional adjustment.

Table S15. The associations of sleep scores, sedentary behavior, and physical activity with cataract after excluding participants with less than 2 years of follow-up.

Supplementary methods

Accelerometer-derived movement behaviors

Between February 2013 and December 2015, 236,519 UK Biobank participants were invited to wear a wrist-worn accelerometer for one week. Of these, 106,053 individuals consented to participate, and complete data on sleep, physical activity (PA), and sedentary behavior (SB) were obtained from 103,636 participants. The processing and analytical methods for accelerometry data have been previously detailed [39]. Briefly, a validated machine learning model, incorporating balanced random forests with Hidden Markov models, was used to classify various movement behaviors in 30-second time windows, including moderate-to-vigorous-intensity physical activity (MVPA), light-intensity physical activity (LIPA), SB, and sleep. We extracted and summarized the total volume of sleep, LIPA, and SB in hours per day, while MVPA was measured in minutes per week.

Inclusion and exclusion of the study population

In this study, we excluded participants with insufficient wear time (<72 h), poor device calibration, or average acceleration values greater than 100 mg, and prevalent cataract cases before the accelerometer completion. Finally, we included 91,127 in the subsample analysis (Supplementary Figure 2).

Table S1. Variable ID and Codes Used for Cataract Diagnosis in the UK Biobank.

Code type	Codes
ICD-10	H250, H251, H252, H258, H259, H261, H262, H263, H264, H268, H269, H280, H281, H282
ICD-9	366, 3661, 3662, 3663, 3664, 3665, 3668, 3669
OPCS4	C71.2, C75.1
Self-reported	5441, 6148, 4700, 5324

ICD, International Classification of Diseases; OPCS: Classification of Surgical Operations and Procedures

Table S2. The scoring system of sleep.

**Characteristics**	**UK BioBank Questionnaire**	**Healthy Answer (%)**	**Unhealthy Answer (%)**
Chronotype	Do you consider yourself to be?	Definitely a "morning" person; More a "morning" than "evening" person.	More an "evening" than a "morning person; Definitely an "evening" person.
Sleep Duration	About how many hours sleep do you get in every 24 hours? (please include naps)	7-8 hr/d	<7 or >=9 hr/d
Insomnia	Do you have trouble falling asleep at night or do you wake up in the middle of the night?	Never/rarely; Sometimes	Usually
Snoring	Does your partner or a close relative or friend complain about your snoring?	No	Yes
Daytime Sleepiness	How likely are you to doze off or fall asleep during the daytime when you don't mean to? (e.g. when working, reading or driving)	Never/rarely; Sometimes	Often; All the Time

Table S3. The resource and definition of the selected covariates (except for age, and sex).

Covariates	UK BioBank Code	Description	Type	Category
Ethnicity	21000	Ethnic background	Categorical	Whites;<br>Non-whites
Socioeconomic status	189	The existing variable 'Townsend area deprivation index' (189) served as an indicator of socioeconomic status, with higher scores indicating greater socioeconomic deprivation.	Continuous	-
Education	6138	Answers were from ACE touchscreen question "Which of the following qualifications do you have? (You can select more than one)". The existing variable ' Qualifications' (6138) was applied.	Categorical	NVQ/CSE/A levels/others;<br>College or university degree;<br>None
Smoking status	20116	The existing variable 'smoking status' (20116) was applied.	Categorical	Never;<br>Previous smoker;<br>Current smoker
Drinking status	20117	The existing variable 'Alcohol drinker status ' (20117) was applied.	Categorical	Never;<br>Previous drinker;<br>Current drinker
Sun exposure	1050	The existing variable ' Time spend outdoors in summer ' (1050) was applied.	Continuous	-
Diabetes	2443, 2976, 6153, 6177, 20002,	Diabetes status was defined as history of diabetes, glucose ≥7.0 mmol/L, or HbA1c ≥6.5%.	Categorical	Yes;<br>No
Hypertension	2966, 6150, 6153, 6177, 20002	Hypertension was defined as history of hypertension.	Categorical	Yes;<br>No

For further information, please refer to the UK Biobank data showcase https://biobank.ndph.ox.ac.uk/showcase/search.cgi

Table S4. The joint associations of sedentary behavior, and physical activity with cataract.

	Cross-sectional associations		Longitudinal associations	
Exposure	OR (95% CI) [a]	P value	HR (95% CI) [a]	P value
**Low SB**				
High PA	1.00	-	1.00	-
Moderate PA	1.07 (0.99 to 1.15)	0.109	1,07 (0.99 to 1.15)	0.042
Low PA	1.00 (0.92 to 1.09)	0.935	1.03 (0.99 to 1.07)	0.189
**Moderate SB**				
High PA	0.99 (0.93 to 1.04)	0.600	1.03 (1.00 to 1.06)	0.042
Moderate PA	1.00 (0.92 to 1.08)	0.990	1.07 (1.03 to 1.11)	0.189
Low PA	1.04 (0.96 to 1.12)	0.343	1.06 (1.02 to 1.10)	0.002
**High SB**				
High PA	1.03 (0.97 to 1.09)	0.357	1.07 (1.04 to 1.10)	<0.001
Moderate PA	1.05 (0.97 to 1.15)	0.236	1.13 (1.08 to 1.18)	<0.001
Low PA	1.14 (1.05 to 1.23)	0.001	1.14 (1.09 to 1.18)	<0.001

[a] Adjusted for age, sex, ethnicity, socioeconomic status, education, body mass index, smoking status, drinking status, sun exposure, diabetes and hypertension.

Abbreviations: OR, odds ratio; HR, hazard ratio; CI, confidence interval; SB, sedentary behavior; PA, physical activity

Table S5. The joint associations of sedentary behavior, and sleep scores with cataract.

	Cross-sectional associations		Longitudinal associations	
Exposure	OR (95% CI) [a]	P value	HR (95% CI) [a]	P value
**Low SB**				
Healthy sleep	1.00	-	1.00	-
Intermediate sleep	1.13 (1.06 to 1.20)	<0.001	1.05 (1.02 to 1.09)	0.001
Poor sleep	1.15 (0.95 to 1.38)	0.152	1.12 (1.02 to 1.23)	0.022
**Moderate SB**				
Healthy sleep	0.99 (0.93 to 1.05)	0.670	1.02 (0.99 to 1.05)	0.116
Intermediate sleep	1.09 (1.02 to 1.15)	0.008	1.10 (1.06 to 1.13)	<0.001
Poor sleep	1.36 (1.16 to 1.59)	<0.001	1.12 (1.03 to 1.22)	0.01
**High SB**				
Healthy sleep	1.03 (0.97 to 1.10)	0.345	1.09 (1.06 to 1.13)	<0.001
Intermediate sleep	1.15 (1.08 to 1.22)	<0.001	1.12 (1.08 to 1.15)	<0.001
Poor sleep	1.51 (1.30 to 1.74)	<0.001	1.26 (1.17 to 1.36)	<0.001

[a] Adjusted for age, sex, ethnicity, socioeconomic status, education, body mass index, smoking status, drinking status, sun exposure, diabetes and hypertension.

Abbreviations: OR, odds ratio; HR, hazard ratio; CI, confidence interval; SB, sedentary behavior

Table S6. The joint associations of sleep scores and physical activity with cataract.

	Cross-sectional associations		Longitudinal associations	
Exposure	OR (95% CI) [a]	P value	HR (95% CI) [a]	P value
**Healthy sleep**				
High PA	1.00	-	1.00	-
Moderate PA	1.05 (0.98 to 1.12)	0.133	1.05 (1.02 to 1.08)	0.003
Low PA	1.05 (0.98 to 1.13)	0.149	1.03 (1.00 to 1.07)	0.049
**Intermediate sleep**				
High PA	1.13 (1.08 to 1.18)	<0.001	1.05 (1.03 to 1.08)	<0.001
Moderate PA	1.14 (1.06 to 1.22)	<0.001	1.10 (1.06 to 1.13)	<0.001
Low PA	1.15 (1.07 to 1.23)	<0.001	1.10 (1.06 to 1.14)	<0.001
**Poor sleep**				
High PA	1.22 (1.06 to 1.39)	0.004	1.11 (1.04 to 1.19)	0.002
Moderate PA	1.37 (1.11 to 1.68)	0.003	1.14 (1.03 to 1.27)	0.015
Low PA	1.68 (1.43 to 1.96)	<0.001	1.23 (1.13 to 1.35)	<0.001

[a] Adjusted for age, sex, ethnicity, socioeconomic status, education, body mass index, smoking status, drinking status, sun exposure, diabetes and hypertension.

Abbreviations: OR, odds ratio; HR, hazard ratio; CI, confidence interval; PA, physical activity

Table S7. Analyses on interaction of sleep scores and physical activity with incident cataract.

	Healthy sleep		Intermediate and poor sleep		Hazard Ratio (95% CI) for sleep scores within strata of PA
	N with/without events	Hazard Ratio (95% CI)	N with/without events	Hazard Ratio (95% CI)	
Ideal PA	5061/35,123	1.02 (0.99, 1.06), P = 0.195	6132/37,460	1.10 (1.06, 1.13), P < 0.001	1.07 (1.03, 1.12), P = 0.001
Non-ideal PA	23,896/168,263	1.00 (Reference)	20,568/130,036	1.05 (1.03, 1.08), P < 0.001	1.06 (1.03, 1.08), P < 0.001
Hazard Ratio (95% CI) for non-ideal PA within strata of sleep		1.02 (0.99, 1.06), P = 0.205		1.04 (1.01, 1.07), P = 0.014	

Multiplicative interaction: HR for product term 1.03, 95% CI 1.00 to1.07; addictive interaction: RERI, -0.13, 95% CI -0.21 to -0.04; AP, -0.12, 95% CI -0.21 to -0.04.

Hazard ratios were adjusted for age, sex, ethnicity, socioeconomic status, education, body mass index, smoking status, drinking status, sun exposure, hypertension and diabetes.

Abbreviations: CI, confidence interval; PA, physical activity

Table S8. Analyses on interaction of sedentary behavior and physical activity with incident cataract.

	Low-to-moderate SB		High SB		Hazard Ratio (95% CI) for high SB within strata of PA
	N with/without events	Hazard Ratio (95% CI)	N with/without events	Hazard Ratio (95% CI)	
Non-ideal PA	7169/48,320	1.02 (0.99, 1.05), P = 0.146	4024/24,263	1.11 (1.07, 1.15), P < 0.001	1.08 (1.04, 1.13), P < 0.001
Ideal PA	32,370/220,900	1.00 (Reference)	12,095/77,399	1.06 (1.03, 1.08), P < 0.001	1.06 (1.03, 1.08), P < 0.001
Hazard Ratio (95% CI) for non-ideal PA within strata of SB		1.02 (1.00, 1.05), P = 0.107		1.05 (1.01, 1.09), P = 0.023	

Multiplicative interaction: HR for product term 1.02, 95% CI 0.98 to1.07; addictive interaction: RERI, -0.08, 95% CI -0.14 to -0.02; AP, -0.08, 95% CI -0.14 to -0.02.

Hazard ratios were adjusted for age, sex, ethnicity, socioeconomic status, education, body mass index, smoking status, drinking status, sun exposure, hypertension and diabetes.

Abbreviations: CI, confidence interval; PA, physical activity; SB, sedentary behavior

Table S9. Analyses on interaction of sleep scores and sedentary behavior with incident cataract.

	Healthy sleep		Intermediate and poor sleep		Hazard Ratio (95% CI) for sleep scores within strata of SB
	N with/without events	Hazard Ratio (95% CI)	N with/without events	Hazard Ratio (95% CI)	
Low-to-moderate SB	21,488/154,843	1.00 (Reference)	18,051/114,377	1.02 (0.99, 1.05), P = 0.146	1.07 (1.04, 1.09), P < 0.001
High SB	7470/48,543	1.06 (1.03, 1.08), P < 0.001	8649/53,119	1.11 (1.07, 1.15), P < 0.001	1.03 (1.00, 1.07), P = 0.049
Hazard Ratio (95% CI) for high SB within strata of sleep		1.08 (1.05, 1.11), P < 0.001		1.04 (1.02, 1.07), P = 0.002	

Multiplicative interaction: HR for product term 1.11, 95% CI 1.01 to 1.23; addictive interaction: RERI, -0.03, 95% CI -0.14 to 0.08; AP, -0.03, 95% CI -0.13 to 0.08.

Hazard ratios were adjusted for age, sex, ethnicity, socioeconomic status, education, body mass index, smoking status, drinking status, sun exposure, hypertension and diabetes.

Abbreviations: CI, confidence interval; SB, sedentary behavior; RERI, Relative Excess Risk due to Interaction; AP, Attributable Proportion due to Interaction

Table S10. Associations of accelerometer-derived sleep and physical activity with incident cataract by replacing 1 hour of sedentary behavior using isotemporal substitution model.

Activity	Model 1 [a]		Model 2 [b]	
	HR (95% CI)	P value	HR (95% CI)	P value
**Whole population**				
LIPA	0.970 (0.957-0.985)	<0.001	0.980 (0.965-0.995)	0.011
MVPA	0.912 (0.876-0.950)	<0.001	0.943 (0.903-0.985)	0.008
Sleep	0.973 (0.956-0.990)	0.002	0.982 (0.964-1.000)	0.049
**Sleep duration ≤ 7 h/day**				
LIPA	0.970 (0.954-0.985)	<0.001	0.980 (0.964-0.997)	0.024
MVPA	0.927 (0.886-0.970)	0.001	0.964 (0.918-1.012)	0.137
Sleep	0.981 (0.950-1.013)	0.244	0.993 (0.960-1.028)	0.693
**Sleep duration > 7 h/day**				
LIPA	0.972 (0.950-0.994)	0.015	0.983 (0.959-1.007)	0.166
MVPA	0.873 (0.820-0.931)	<0.001	0.908 (0.849-0.972)	0.005
Sleep	0.990 (0.958-1.022)	0.525	0.993 (0.960-1.027)	0.688

[a] Adjusted for age and sex.

[b] Adjusted for age, sex, ethnicity, socioeconomic status, education, body mass index, smoking status, alcohol consumption, sun exposure, diabetes and hypertension.

Abbreviations: HR, hazard ratio; CI, confidence interval; SB, sedentary behavior; LIPA, light intensity of physical activity; MVPA, moderate-to-vigorous intensity of physical activity

Table S11. The longitudinal associations of sleep scores, sedentary behavior, and physical activity with cataract stratified by age.

Exposure	HR (95% CI)		HR (95% CI)	
	Model 1[a]	P value	Model 2[b]	P value
**Elderly**				
**Sleep pattern**				
Poor	1.19 (1.13 to 1.26)	<0.001	1.08 (1.02 to 1.15)	0.012
Intermediate	1.08 (1.06 to 1.10)	<0.001	1.04 (1.02 to 1.07)	<0.001
Healthy	1.00			
**Sedentary behavior**				
High	1.13 (1.10 to 1.15)	<0.001	1.08 (1.05 to 1.11)	<0.001
Moderate	1.03 (1.01 to 1.06)	0.004	1.02 (1.00 to 1.05)	0.1
Low	1.00			
**Physical activity**				
Low	1.09 (1.06 to 1.12)	<0.001	1.04 (1.01 to 1.07)	0.008
Moderate	1.07 (1.04 to 1.10)	<0.001	1.05 (1.02 to 1.08)	<0.001
High	1.00			
**Midlife**				
**Sleep pattern**				
Poor	1.41 (1.30 to 1.53)	<0.001	1.22 (1.12 to 1.33)	<0.001
Intermediate	1.12 (1.09 to 1.16)	<0.001	1.07 (1.04 to 1.11)	<0.001
Healthy	1.00			
**Sedentary behavior**				
High	1.14 (1.10 to 1.19)	<0.001	1.10 (1.05 to 1.15)	<0.001
Moderate	1.07 (1.04 to 1.11)	<0.001	1.06 (1.02 to 1.10)	0.003
Low	1.00			
**Physical activity**				
Low	1.13 (1.09 to 1.18)	<0.001	1.04 (1.00 to 1.09)	0.038
Moderate	1.06 (1.02 to 1.10)	0.006	1.02 (0.98 to 1.07)	0.255
High	1.00			

[a] Adjusted for age and sex.

[b] Further adjusted for ethnicity, socioeconomic status, education, body mass index, smoking status, drinking status, sun exposure, diabetes and hypertension.

Abbreviations: HR, hazard ratio; CI, confidence interval

Table S12. The longitudinal associations of sleep scores, sedentary behavior, and physical activity with cataract stratified by sex.

Exposure	HR (95% CI)		HR (95% CI)	
	Model 1[a]	P value	Model 2[b]	P value
**Female**				
**Sleep pattern**				
Poor	1.26 (1.19 to 1.34)	<0.001	1.15 (1.08 to 1.23)	<0.001
Intermediate	1.09 (1.07 to 1.12)	<0.001	1.05 (1.03 to 1.08)	<0.001
Healthy	1.00			
**Sedentary behavior**				
High	1.15 (1.12 to 1.18)	<0.001	1.10 (1.07 to 1.14)	<0.001
Moderate	1.05 (1.02 to 1.07)	<0.001	1.03 (1.00 to 1.06)	0.023
Low	1.00			
**Physical activity**				
Low	1.07 (1.04 to 1.10)	<0.001	1.02 (0.99 to 1.06)	0.117
Moderate	1.05 (1.02 to 1.08)	0.001	1.04 (1.00 to 1.07)	0.023
High	1.00			
**Male**				
**Sleep pattern**				
Poor	1.27 (1.18 to 1.36)	<0.001	1.11 (1.03 to 1.20)	0.005
Intermediate	1.09 (1.06 to 1.12)	<0.001	1.05(1.03 to 1.08)	<0.001
Healthy	1.00			
**Sedentary behavior**				
High	1.11 (1.07 to 1.14)	<0.001	1.06 (1.02 to 1.10)	0.001
Moderate	1.04 (1.00 to 1.07)	0.027	1.03 (0.99 to 1.06)	0.122
Low	1.00			
**Physical activity**				
Low	1.15 (1.11 to 1.19)	<0.001	1.08 (1.04 to 1.12)	<0.001
Moderate	1.10 (1.06 to 1.14)	<0.001	1.07 (1.03 to 1.10)	<0.001
High	1.00			

[a] Adjusted for age.

[b] Further adjusted for ethnicity, socioeconomic status, education, body mass index, smoking status, drinking status, sun exposure, diabetes and hypertension.

Abbreviations: HR, hazard ratio; CI, confidence interval

Table S13. The longitudinal associations of sleep scores, sedentary behavior, and physical activity with cataract stratified by diabetes.

Exposure	HR (95% CI)		HR (95% CI)	
	Model 1[a]	P value	Model 2[b]	P value
**Diabetes**				
**Sleep pattern**				
Poor	1.17 (1.07 to 1.28)	0.001	1.15 (1.04 to 1.27)	0.007
Intermediate	1.05 (1.01 to 1.10)	0.017	1.05 (1.01 to 1.10)	0.028
Healthy	1.00			
**Sedentary behavior**				
High	1.05 (0.99 to 1.11)	0.101	1.07 (1.00 to 1.13)	0.035
Moderate	1.01 (0.95 to 1.06)	0.852	1.02 (0.97 to 1.09)	0.43
Low	1.00			
**Physical activity**				
Low	1.08 (1.03 to 1.14)	0.002	1.05 (0.99 to 1.11)	0.11
Moderate	1.11 (1.05 to 1.17)	<0.001	1.09 (1.03 to 1.16)	0.004
High	1.00			
**Non-diabetes**				
**Sleep pattern**				
Poor	1.19 (1.12 to 1.25)	<0.001	1.12 (1.06 to 1.19)	<0.001
Intermediate	1.07 (1.05 to 1.09)	<0.001	1.05 (1.03 to 1.07)	<0.001
Healthy	1.00			
**Sedentary behavior**				
High	1.09 (1.07 to 1.12)	<0.001	1.09 (1.06 to 1.12)	<0.001
Moderate	1.03 (1.01 to 1.05)	0.003	1.03 (1.01 to 1.06)	0.004
Low	1.00			
**Physical activity**				
Low	1.07 (1.05 to 1.10)	<0.001	1.04 (1.02 to 1.07)	0.001
Moderate	1.05 (1.03 to 1.08)	<0.001	1.04 (1.01 to 1.07)	0.003
High	1.00			

[a] Adjusted for age and sex.

[b] Further adjusted for ethnicity, socioeconomic status, education, body mass index, smoking status, drinking status, sun exposure, and hypertension.

Abbreviations: HR, hazard ratio; CI, confidence interval

Table S14. The longitudinal associations between sleep scores, sedentary behavior, and physical activity and cataract with additional adjustment.

Exposure	HR (95% CI)			
	Model 3 [a]	Model 4 [b]	Model 5 [c]	Model 6 [d]
**Sleep pattern**				
Poor	1.13 (1.07 to 1.18)	1.16 (1.10, 1.22)	1.11 (1.06, 1.17)	1.12 (1.06, 1.18)
Intermediate	1.05 (1.03 to 1.07)	1.06 (1.04, 1.08)	1.05 (1.03, 1.07)	1.05 (1.03, 1.07)
Healthy	1.00	1.00	1.00	1.00
**Sedentary behavior**				
High	1.07 (1.05 to 1.10)	1.08 (1.06, 1.11)	1.09 (1.06, 1.11)	1.08 (1.05, 1.11)
Moderate	1.03 (1.01 to 1.05)	1.03 (1.01, 1.05)	1.04 (1.01, 1.06)	1.04 (1.01, 1.06)
Low	1.00	1.00	1.00	1.00
**Physical activity**				
Low	1.04 (1.01 to 1.06)	1.05 (1.02, 1.08)	1.04 (1.02, 1.07)	1.05 (1.02, 1.08)
Moderate	1.04 (1.02 to 1.07)	1.05 (1.02, 1.08)	1.06 (1.03, 1.08)	1.05 (1.03, 1.08)
High	1.00	1.00	1.00	1.00

[a] Further adjusted for physical activity and sedentary behavior (in sleep pattern), sleep and physical activity (in sedentary behavior), or sleep and sedentary behavior (in physical activity) in addition to covariates in model 2.

[b] Further adjusted for myopia in addition to covariates in model 2.

[c] Further adjusted for eye trauma in addition to covariates in model 2.

[d] Further adjusted for use of corticosteroids in addition to covariates in model 2.

Abbreviations: HR, hazard ratio; CI, confidence interval

Table S15. The associations of sleep scores, sedentary behavior, and physical activity with cataract after excluding participants with less than 2 years of follow-up.

	HR (95% CI)		HR (95% CI)	
Exposure	Model 1[a]	P value	Model 2[b]	P value
**Sleep pattern**				
Poor	1.27 (1.21 to1.33)	<0.001	1.15 (1.09 to 1.20)	<0.001
Intermediate	1.09 (1.08 to1.11)	<0.001	1.06 (1.04 to 1.08)	<0.001
Healthy	1.00		1.00	
**Sedentary behavior**				
High	1.13 (1.11 to1.16)	<0.001	1.08 (1.06 to 1.11)	<0.001
Moderate	1.05 (1.03 to1.07)	<0.001	1.04 (1.01 to 1.06)	0.002
Low	1.00		1.00	
**Physical activity**				
Low	1.10 (1.08 to1.12)	<0.001	1.04 (1.02 to 1.07)	0.001
Moderate	1.07 (1.05 to1.10)	<0.001	1.05 (1.02 to 1.07)	<0.001
High	1.00		1.00	

[a] Adjusted for age and sex.

[b] Further adjusted for ethnicity, socioeconomic status, education, body mass index, smoking status, drinking status, sun exposure, diabetes and hypertension.

Abbreviations: HR, hazard ratio; CI, confidence interval
